# ADVANCE CARE PLANNING AND END-OF-LIFE EXPERIENCES OF HIGH HEALTH CARE UTILIZERS IN THE LAST YEAR OF LIFE

**DOI:** 10.1093/geroni/igae098.2635

**Published:** 2024-12-31

**Authors:** Omar Marin, Sriya Bandi, Charlotte Duty, Alex Tate

**Affiliations:** University of Chicago, Chicago, Illinois, United States; University of Chicago, Chicago, Illinois, United States; University of Chicago, Chicago, Illinois, United States; University of Chicago, Chicago, Illinois, United States

## Abstract

As the U.S. population continues to get older, Americans are projected to consume more healthcare than ever before. Advance care planning (ACP), which documents future goals of care in the context of limited decision-making capacity, is essential to delivering patient-centered care aligned with wishes and values and correlates with less utilization at the end-of-life (EOL). ACP is critical for high healthcare utilizers, who have multiple comorbid conditions and often unmet social needs. This pilot study used proxy postmortem interviews (n=20) to identify and explicate the most common factors shaping the EOL care experiences of high healthcare utilizers dwelling in vulnerable Chicago neighborhoods, and ACP’s role in these experiences. Modified open coding of data revealed five central themes in shaping decedents’ EOL experiences and ACP: (1) decedent support from immediate network ties, (2) patient acceptance of declining health, (3) proxy confidence in EOL decision making, (4) individual values motivating pursuing certain types of care, and (5) an orientation towards the treatment imperative, defined by Shim et al. (2008) as an inexorable momentum towards treatment at any cost. Within the last 12 months of life, even if patients had a formal or informal ACP in place, proxies reported a default to more medical intervention by patients. Just five proxies reported care transitions to palliative/hospice care. This pilot study presents a novel approach to better understanding EOL care and experiences of vulnerable high healthcare utilizers and underscores a need for further investigation into the relationship between ACP and EOL experiences among this group.

